# The relationship between the perception of open disclosure of patient safety incidents, perception of patient safety culture, and ethical awareness in nurses

**DOI:** 10.1186/s12910-020-00546-7

**Published:** 2020-10-27

**Authors:** Yujeong Kim, Eunmi Lee

**Affiliations:** 1 College of Nursing, Research Institute of Nursing Science, Kyungpook National University, 680 Gukchabosangro, Jung-gu, Daegu, 41944 Republic of Korea; 2 Department of Nursing, Research Institute for Basic Science, Hoseo University, 20, Hoseo-ro79beon-gil, Baebang-eup, Asan-si, Chungcheongnam-do 31499 Republic of Korea

**Keywords:** Disclosure, Patient safety, Ethical awareness, Principle-based ethics

## Abstract

**Background:**

Scientific advances have resulted in more complex medical systems, which in turn have led to an increase in the number of patient safety incidents (PSIs). In this environment, the importance of honest disclosure of PSIs is rising, which highlight the need to settle a reliable system. This study aimed to investigate the effects of patient safety culture and ethical awareness on open disclosure of PSIs.

**Methods:**

Data were collected from 389 nurses using self-reported perceptions of open disclosure of PSIs, perceptions of patient safety culture, and ethical awareness.

**Results:**

Perception of open disclosure of PSIs was significantly correlated with ethical awareness and perception of patient safety culture. Ethical awareness had the greatest impact on perception of PSIs, and two components of the perception of patient safety culture, namely overall knowledge about patient safety and staffing, were found to have significant effects.

**Conclusions:**

To enhance nurses’ perception of open disclosure of PSIs, educational curriculum and programs that teach and practice fundamental ethical values are needed. Furthermore, it also calls for effort on the part of healthcare institutions and the government, as well as people’s trust, to implement a legal safety net and foster patient safety culture to promote honest disclosure of PSIs to patients.

## Background

Although scientific advances have increased the outcome of medicine, they have also resulted in more specialized and complex medical systems, which have in turn led to more patient safety incidents (PSIs). In fact, one out of every ten inpatients suffer an unintentional injury not related to their disease at the hospital, and half of these cases are reported to have been preventable [1]. In countries such as the United States, Canada, and Australia, healthcare institutions prioritize patient safety and candidly report all PSIs in order to ameliorate them. As part of such efforts, these countries have enacted healthcare accreditation criteria for patient safety, obligating relevant institutions to disclose incident outcomes to the public [2–4]. In particular, these countries implemented open disclosure programs for PSIs, where healthcare professionals and hospitals not only express regret about an incident to patients and guardians, but also provide factual accounts of the incident, explain the outcomes of incident analysis, and describe measures to prevent similar incidents—whether they are at fault or not [5].

According to recent studies, open disclosures of PSIs help to maintain a trusting relationship between healthcare professionals and patients and to cut costs of medical malpractice claims and payouts [6, 7]. The University of Michigan Health System reported that its medical malpractice claims were halved and annual litigation costs cut by one-third since the implementation of an open disclosure program, and that 5 years after its implementation, 98% of its healthcare professionals support the program and report that the program is a major factor for their continued practice at the corresponding hospital [5].

According to health law, healthcare professionals have a legal responsibility to patients based on the principles of beneficence and nonmaleficence; this makes it difficult to practice open disclosure since expression of regret can be seen as admission of guilt—even before personal fault has been confirmed. Thus, the United States has enacted the apology law to protect healthcare professionals, where their disclosures cannot be used as legal evidence against them in court. In Australia, the government implemented a standardized guideline to help healthcare professionals communicate PSIs without causing a misunderstanding with the patient. Many other healthcare institutions have implemented relevant programs [6–8]. Meanwhile, it has been reported that Korea has a different cultural perception of apology as compared with other Western countries. According to a study comparing the cultural and social implications of apology among Koreans and Americans [9], it has been reported that on the one hand, Americans consider apology as a kind of good manner characterized by seeking forgiveness while simultaneously restoring a damaged image or reputation; on the other hand, Koreans believe that an apology only has the effect of seeking forgiveness, which sometimes threatens their honor. In addition, it has been reported that Koreans, given their collectivistic culture, believe that it is desirable to apologize in a crisis situation and accept the fault generously because of their strong tendency to attribute failure to themselves and not to others [10]. Therefore, the perception regarding apology and open disclosure of PSIs may differ across cultures.

In Korea, the Patient Safety Act was announced in July 2016. Healthcare institutions have since appointed an employee in charge of patient safety to facilitate the reporting of PSIs. However, the full practice of open disclosure remains limited: only its rationale was discussed in some conferences on patient safety. Consider nurses—healthcare professionals with the most frequent contact with patients and who possibly develop close relationships with many of them. They experience a number of diverse PSIs frequently, including medication error, falls, and bedsores. Once an incident occurs, the nurse responsible agonizes over how to explain the incident to the patient and caregiver, and suffers stress and conflicting emotions when reporting the PSI to the physician and head nurse [11]. It would be useful to investigate how nurses perceive open disclosure of PSIs; from there, we can identify ways to help nurses to practice open disclosure while maintaining trust from patients and caregivers.

Not many studies in Korea have examined how nurses perceive disclosure of PSIs. Some international qualitative studies on nurses reported that it is difficult to define error when disclosing PSIs, and the perception and degree of disclosure of PSIs vary according to patient characteristics, nurses’ ethical awareness and fear, and patient safety culture in the corresponding healthcare institution [12]. Ethical awareness is a perception of the inherently ethical nature of nursing practice that enables nurses to recognize the ethical implications of all practice actions [13]. Because health delivery systems and laws differ across countries, patient safety culture and nurses’ ethical awareness of disclosure may differ. A study reported that Korean nurses experienced taking the blame as unfair when disclosing PSIs, and felt that patient safety culture was still at an inchoate stage [14]. Thus, this study aims to investigate the effects of patient safety culture (organizational characteristic) and nurses’ ethical awareness (individual characteristic) on Korean nurses’ perceptions of open disclosure of PSIs. Based on the findings, we attempt to lay a foundation to institutionalize open disclosure of PSIs in Korea’s healthcare industry.

## Methods

### Design

This study uses a cross-sectional survey to examine the relationships between the following variables: perceptions of open disclosure of PSIs, perceptions of patient safety culture, and ethical awareness in nurses.

### Study population

Data were collected from nurses in general hospitals or tertiary hospitals in Korea from July 30 to September 31, 2018. We performed convenience sampling by recruiting participants via bulletin board announcements that were put up at 25 hospitals. The participants voluntarily signed a form for informed consent. They were instructed to drop the questionnaire into a collection box after completing the survey. It took about ten minutes for each person to complete the survey. The appropriate sample size required for regression analysis was computed using the G*Power 3.1.9.2 software. For an effect size (f2) of 0.02, significance (α) of 0.05, and power (1 − β) of 0.80, the minimum required sample size was 395, but we distributed the questionnaire to 420 nurses in consideration of potential withdrawal. A total of 393 questionnaires were retrieved, and after excluding four questionnaires for having incomplete responses, a total of 389 were included in the final analysis.

### Measures

#### General characteristics

We collected information about participants’ sex, age, education level, length of nursing career, current position, type of healthcare institution, and working unit/area.

#### Perception of open disclosure of PSIs

Nurses’ perception of open disclosure of PSIs [15] was assessed using a questionnaire modified and adapted by us based on the study on nurses in nursing homes by Wagner et al. [16]; the study on physicians by Kadjian et al. [17]; and the systematic review on open disclosure of PSIs by Lee et al. [18]. The contents of the survey were assessed by a panel of four experts, including one professor of a patient safety association and three nurses specializing in patient safety with a career of five years or longer. The content validity index (CVI) was computed based on the experts’ ratings, and the S-CVI was 0.89. A pilot test was conducted on five convenience sample nurses in a general hospital or tertiary hospital. The survey comprised 30 items in six domains: open disclosure across harm level (3 items), open disclosure across situation (6 items), justification of open disclosure (4 items), negative consequences of open disclosure (5 items), positive consequences of open disclosure (6 items), and facilitators of open disclosure (6 items). The open disclosure across harm level domain comprises items (no harm, minor harm, and severe harm) on whether patients and their families should be notified regarding PSIs. The open disclosure across situation domain comprises items on whether nurses should initiate open disclosure according to the needs, benefit, or understanding of patients and their families. The justification of open disclosure domain comprises items on whether nurses should initiate open disclosure even if they suffer damages or disadvantages due to it. The negative consequences of open disclosure domain comprises items on whether nurses believe that there will be litigation, damage to reputation, or criticism caused by the disclosure. The positive consequences of open disclosure domain comprises items on whether nurses believe that patient’s trust and medical staff's patient safety interest will increase due to disclosure. Finally, the facilitators of open disclosure domain comprises items on whether nurses believe that ethical awareness, education, support systems, and patient safety culture can promote open disclosure. Each item is rated on a four-point Likert scale, from 1 (*strongly disagree*) to 4 (*strongly agree*). Negatively phrased items were reverse coded. A higher score indicates a higher perception of open disclosure of PSIs. The reliability, as measured with Cronbach’s α, was 0.895.

#### Ethical awareness

Ethical awareness was assessed using the nurses’ ethical awareness scale developed by Jang [19]. This is a 30-item tool based on the Code of Ethics for Korean nurses, with 10 items each about patients, professional work, and partners. This tool includes the contents of nurses’ daily duties, responsibilities, and conflict situations. The tool has 16 positively-worded items and 14 negatively-worded items; the latter type was reverse-scored. Each item is rated on a four-point Likert scale, from 1 (*strongly disagree*) to 4 (*strongly agree*). A higher score indicates a higher moral sensitivity. The Cronbach’s α was 0.71 at the time of development and 0.757 in our study.

#### Perception of patient safety culture

Perception of patient safety culture was assessed using the Hospital Survey on Patient Safety Culture, which was developed by Agency for Healthcare Research and Quality (AHRQ) [20] and adapted by Kim et al. [21]. This 42-item questionnaire consists of 12 Patient Safety Culture Composite (teamwork within units, supervisor/manager expectations and actions promoting patient safety, organizational learning/continuous improvement, management support for patient safety, overall perceptions of patient safety, feedback and communication about error, communication openness, frequency of events reported, teamwork across units, staffing, handoffs and transitions, nonpunitive response to error). Each item is rated on a five-point Likert scale from 1 (*strongly disagree*) to 5 (*strongly agree*), and negatively worded items are reverse scored. A higher score indicates a better attitude toward patient safety. The internal consistency reliability (Cronbach's α) was 0.63–0.84 at the time of development, and that in this study was 0.880.

### Statistical Analysis

To investigate the factors that affect the perception of open disclosure of PSIs in nurses, we first examined control variables among the participants. Differences in perception of open disclosure according to general characteristics (sex, age, education level, length of nursing career, current position, type of healthcare institution, and working unit/area) were analyzed using independent sample t-tests and one-way ANOVA. Difference in perception of open disclosure according to age and length of employment was analyzed with simple regression. Each participant’s perception of patient safety culture was computed based on the proportion of positive responses (4 and 5) in the entire survey after applying the AHRQ method. Correlations among perception of open disclosure, perception of patient safety culture, and ethical awareness were analyzed. To identify the factors that affect the perception of open disclosure, multiple regression analysis was performed with perception of open disclosure as the dependent variable, perception of patient safety culture and ethical awareness as the independent variables, and general characteristics that differed according to the perception of open disclosure by category as the control variables.

## Results

### Participants’ general characteristics

A total of 389 participants were enrolled, 365 (93.8%) of whom were women. The mean age was 35.47 years, and 211 (54.2%) had a bachelor’s degree. The mean length of nursing career was 11.73 years. A total of 224 (57.6%) participants were staff nurses, and 306 (78.7%) of the participants currently work in a general hospital. 170 (43.7%) nurses currently work in a ward while 110 (28.3%) work in the intensive care unit (Table 1).

**Table 1:** Differences of perception of open disclosure of PSIs according to general characteristics (*N* = 389)

Categories	n (%) Mean ± SD	Perception of open disclosure of PSIs	
		Mean ± SD, slope	t (*p*), F (*p*)
Gender			
Male	24 (6.2)	3.00 ± 0.18	− 0.64 (.527)
Female	365 (93.8)	3.02 ± 0.34	
Age	35.47 ± 8.28	0.003	1.39 (.166)
Education			
Associate degree	110 (28.3)	3.03 ± 0.32	0.47 (.625)
Bachelor’s degree	211 (54.2)	3.04 ± 0.33	
Master’s degree or higher	68 (17.5)	2.99 ± 0.39	
Length of nursing career	11.73 ± 9.21	0.001	0.60 (.548)
Position			
Staff nurse	224 (57.6)	3.01 ± 0.33	0.96 (.382)
Head nurse	80 (20.6)	3.07 ± 0.35	
Nurse manager or higher	85 (21.9)	3.03 ± 0.35	
Type of healthcare institution			
General hospital	306 (78.7)	3.02 ± 0.33	− 0.77 (.444)
Tertiary hospital	83 (21.3)	3.05 ± 0.35	
Working unit			
Ward	170 (43.7)	2.99 ± 0.34	0.68 (.642)
Intensive care unit	110 (28.3)	2.96 ± 0.32	
Emergency room	24 (6.2)	2.97 ± 0.22	
OR, recovery room	22 (5.7)	2.97 ± 0.39	
Outpatient clinic, laboratory	25 (6.4)	3.03 ± 0.26	
Administration	38 (9.8)	3.03 ± 0.34	

### Differences in the perception of open disclosure of PSIs according to general characteristics

To examine the relationship between the control variables and dependent variable, the differences in perception of open disclosure according to general characteristics were analyzed. There were no statistically significant differences according to gender, age, education level, length of nursing career, position, type of healthcare institution, and working unit/area (Table 1).

### Relationships among perception of open disclosure of PSI, perception of patient safety culture, and ethical awareness

The mean score for perception of open disclosure was 3.03 (out of 4), and the mean score for ethical awareness was 2.78 (out of 4). Among the components of perception of patient safety culture, the mean score was the highest for “teamwork within units” (3.58 out of 5) followed by “feedback & communication about error” (3.38 out of 5). Components with a score of below 3 were “management support for patient safety” (2.94), “frequency of events reported” (2.88), “staffing” (2.70), and “nonpunitive response to errors” (2.67).

Perception of open disclosure had a statistically significant positive correlation with ethical awareness (*r* = 0.44, *p* < 0.001), and the “teamwork within units” (*r* = 0.10, *p* = 0.023), “organizational learning/continuous improvement” (*r* = 0.13, *p* = 0.005), “overall perceptions of patient safety” (*r* = 0.18, *p* < 0.001), and “staffing” (*r* = − 0.11, *p* = 0.019) components of perception of patient safety culture. Ethical awareness had no statistically significant correlation with the “management support for patient safety,” “teamwork across units,” “staffing,” and “nonpunitive response to error” components of perception of patient safety culture, but did have a statistically significant correlation with all the remaining factors. Most of the perception of patient safety culture had statistically significant positive correlations with their components. However, “staffing” had no statistically significant correlations with ethical awareness and the “teamwork within units,” “organizational learning/continuous improvement,” and “frequency of events reported” components of perception of patient safety culture (Table 2).

**Table 2:** Correlation of perception of open disclosure of PSIs, ethical awareness, and patient safety culture (N = 389)

	Mean ± SD	Perception of open disclosure of PSIs	Ethical awareness	Patient safety culture											
				A	B	C	D	E	F	G	H	I	J	K	L
Perception of open disclosure of PSIs	3.03 ± 0.34														
Ethical awareness	2.78 ± 0.23	.44 (< .001)													
Patient safety culture	A	3.58 ± 0.68	.10 (.023)	.15 (.001)											
	B	3.27 ± 0.66	− .05 (.182)	− .09 (.034)	.12 (.011)										
	C	3.18 ± 0.69	.13 (.005)	.18 (< .001)	.55 (< .001)	.12 (.010)									
	D	2.94 ± 0.58	− .01 (.389)	.04 (.205)	.33 (< .001)	.19 (< .001)	.41 (< .001)								
	E	3.18 ± 0.51	.18 (< .001)	.13 (.007)	.21 (< .001)	.15 (.002)	.33 (< .001)	.26 (< .001)							
	F	3.38 ± 0.66	.04 (.205)	.16 (.001)	.48 (< .001)	.20 (< .001)	.55 (< .001)	.42 (< .001)	.28 (< .001)						
	G	3.13 ± 0.63	.01 (.439)	.14 (.003)	.42 (< .001)	.23 (< .001)	.44 (< .001)	.42 (< .001)	.25 (< .001)	.66 (< .001)					
	H	2.88 ± 0.93	.05 (.168)	.19 (< .001)	.29 (< .001)	.15 (.001)	.39 (< .001)	.38 (< .001)	.17 (.001)	.48 (< .001)	.45 (< .001)				
	I	3.09 ± 0.51	− .01 (.411)	.07 (.082)	.31 (< .001)	.28 (< .001)	.30 (< .001)	.55 (< .001)	.33 (< .001)	.37 (< .001)	.36 (< .001)	.26 (< .001)			
	J	2.70 ± 0.61	− .11 (.019)	− .08 (.071)	− .02 (.381)	.15 (.002)	− .02 (.341)	.13 (.006)	.25 (< .001)	.06 (.119)	.16 (.001)	.04 (.204)	.23 (< .001)		
	K	3.04 ± 0.50	− 01 (.489)	.09 (.043)	.23 (< .001)	.26 (< .001)	.27 (< .001)	.52 (< .001)	.20 (< .001)	.30 (< .001)	.32 (< .001)	.21 (< .001)	.64 (< .001)	.17 (< .001)	
	L	2.67 ± 0.62	− 04 (.196)	.01 (.391)	.17 (.001)	.14 (.002)	.27 (< .001)	.20 (< .001)	.21 (< .001)	.23 (< .001)	.27 (< .001)	.28 (< .001)	.19 (< .001)	.31 (< .001)	.22 (< .001)

A, teamwork within units; B, supervisor/manager expectations and actions promoting patient safety; C, organizational learning/continuous improvement; D, management support for patient safety; E, overall perceptions of patient safety; F, feedback and communication about error; G, communication openness; H, frequency of events reported; I, teamwork across units; J, staffing; K, handoffs and transitions; L, nonpunitive response to error

### Factors affecting perception of open disclosure of PSIs

The factors that affect nurses’ perception of open disclosure of PSIs were identified using multiple regression. The general characteristics shown in Table 1 had no statistically significant effects on the perception of open disclosure of PSIs, so none of them were applied as control variables. The results showed that ethical awareness and the “overall perceptions of patient safety” and “staffing” components of perception of patient safety culture had statistically significant associations (*F* = 8.77, *p* < 0.001, *R*^2^ = 0.235). Ethical awareness had the greatest impact on the perception of open disclosure (Table 3).

**Table 3:** Multiple regression analysis of perception of open disclosure of PSIs (*N* = 389)

Variables		B	S.E.	β	t (p)	R^2^	F (p)
Intercept		1.02	0.23		5.13 (< .001)	.235	8.77 (< .001)
Ethical awareness		0.62	0.07	0.42	8.73 (< .001)		
Patient safety culture	A	0.02	0.03	0.05	0.81 (.417)		
	B	0.01	0.03	0.01	0.24 (.810)		
	C	0.02	0.03	0.04	0.64 (.521)		
	D	− 0.01	0.04	− 0.02	− 0.38 (.705)		
	E	0.12	0.03	0.18	3.40 (.001)		
	F	− 0.20	0.04	− 0.04	− 0.55 (.581)		
	G	− 0.04	0.04	− 0.07	− 1.03 (.305)		
	H	− 0.01	0.02	− 0.03	− 0.59 (.556)		
	I	− 0.04	0.04	− 0.05	− 0.82 (.413)		
	J	− 0.06	0.03	− 0.11	− 2.12 (.035)		
	K	− 0.01	0.04	− 0.01	− 0.06 (.952)		
	L	0.04	0.03	0.07	1.28 (.202)		

A, teamwork within units; B, supervisor/manager expectations and actions promoting patient safety; C, organizational learning—continuous improvement; D, management support for patient safety; E, overall perceptions of patient safety; F, feedback and communication about error; G, communication openness; H, frequency of events reported; I, teamwork across units; J, staffing; K, handoffs and transitions; L, nonpunitive response to error

## Discussion

This study examined the relationships among the variables of perception of open disclosure of PSIs, perception of patient safety culture, and ethical awareness in nurses of general hospitals or tertiary hospitals in Korea. Among the participants of this study, 57.6% were staff nurses while 42.5% were nurse managers. Their working units/areas ranged widely, from general wards to special units, and the mean length of nursing career was 11.7 years. Therefore, we were able to recruit a broad spectrum of participants, but there were no differences in the perception of open disclosures of PSIs according to nurses’ general characteristics and work-related characteristics.

The perception of open disclosure of PSIs was positively correlated with ethical awareness. This is similar to previous findings that emphasized the fundamentals and importance of ethics by shedding light on ethically tense situations in the emergency department, which features a high incidence of PSIs and high significance of openly disclosing PSIs [22]. According to Kant’s theory, lying by healthcare professionals is morally unacceptable, and regulations that strictly obligate them to be honest with patients need to be implemented [23]. However, healthcare professionals, including nurses, are faced with ethical dilemmas when practicing open disclosure: they risk litigation, loss of relationship with patient, fear of hurting one’s own reputation, lack of support from the healthcare institution; they lack communication training to conduct effective disclosure [24]. If nurses withhold PSIs, the error will not be addressed; thus, it would be difficult for the concerned parties to improve, and other nurses would likely repeat the same mistake. The perception of open disclosure of PSIs was significantly correlated with the teamwork within units, organizational learning/continuous improvement, overall perceptions of patient safety, and staffing components of the perception of patient safety culture. This is in line with previous results that reported that inadequate staffing, information, and education hinder efforts to improve the perception of PSIs and implement patient safety programs [25]. Therefore, fostering a patient safety culture in healthcare institutions is a pressing matter—it promotes positive perception of open disclosure and helps healthcare professionals to practice it. By acknowledging safety management and patient identification as important factors in preventing PSIs (in accident-prone areas in the course of care), the certification system will impact nurses’ patient safety awareness [26]. If we can institutionalize a system within the hospital to identify the causes of PSIs, we can reduce the recurrence of errors [27]. Moreover, to establish patient safety culture, hospitals must work hard to initiate patient safety management procedures or systems in the ward to secure the necessary evidence for improvement in the event of PSIs. Further, it is also essential to establish regulations and guidelines for open disclosure of PSIs, and to educate and train healthcare professionals. The importance of nursing ethics and patient safety should be continuously emphasized and taught through the nursing undergraduate curriculum. However, in Korea, culture and awareness have not been widely adopted in relation to patient safety cases. Therefore, to raise ethical awareness among nurses and awareness of the patient safety culture in PSIs, it is necessary to continuously provide a culture of education, awareness, and practice of essential ethical values.

Ethical awareness was found to have the most potent impact on the perception of open disclosure of PSIs. Ethical principles such as autonomy, patient's right to know, respect for humanity, transparency, honesty, trust, loyalty, good deeds, and undesirable behavior can be applied to disclosure of PSIs [28]; thus, as shown in the results of this study, ethical awareness may be closely correlated with perception of open disclosure of PSIs. However, perception of open disclosure of PSIs may not be directly related to the actual intention for disclosure of PSIs. Nurses are aware that acknowledging their mistakes when disclosing nursing error is the right thing to do [29]. However, it has been reported that nurses face the reality of being unable to disclose PSIs due to reasons such as immature patient safety culture, lack of resources for protection and support of disclosure, and events with unknown causes [14]. Especially, honest disclosure is difficult in Korea amid lack of any legal regulations or protection for healthcare professionals in the processing of medical malpractice. That said, as time goes by, hospitals would have to take up the people’s demands for transparency, as did enterprises and the government; concealing or remaining silent about an error or malpractice is becoming increasingly difficult. It has been reported that what patients and caregivers want are explanations for the incident, measures to prevent future incidents, and a sincere apology [24]. It is important to gain the patient’s trust by being truthful and forthcoming in explaining how the hospital will deal with the incident. An explanation is the beginning of crisis management, and providing a thorough explanation in the early stage helps the hospital earn more trust from the patient. Nurses must not be financially punished or be pressed by the management from their honest and ethical disclosure of a PSI. Open disclosure of PSIs could be further normalized only with the enactment of legal safety nets that prohibit the use of healthcare professionals’ apologies against them in court, such as the apology law or disclosure law. The apology law stipulates that any expressions of sympathy, regret, or apology used by a healthcare professional during the disclosure of a PSI is not viewed as an admission of legal responsibility in court [30]; this law has been enacted in about 30 US states. Such legal systems not only lower the psychological burden of each healthcare professional but also can cultivate more positive views towards open disclosure of PSIs. Further, it is also necessary to initiate open disclosure programs and introduce success stories to healthcare professionals and the public so as to foster a clearer patient safety culture.

The overall perceptions of patient safety and staffing components of perception of patient safety culture were found to have significant effects on the perception of open disclosure of PSIs. This shows the need to improve nurses’ perception of patient safety culture as well as the need for additional staffing [29, 30]. Developed countries, including the United States, argue that it is important to not only improve nurses’ perceptions about patient safety through education about ethical awareness, patient safety, and communication but also improve the organization, implement continuous and systematic accreditation program, and create a safe environment, and these countries have carried out relevant measures [31]. The most important thing is to systematize how healthcare professionals should communicate a PSI and foster an organizational culture that promotes healthcare professionals, including nurses, to effectively communicate with patients and caregivers. Patient safety and the quality of healthcare service are influenced by nursing staffing and quality [32]. Moreover, an inadequate nursing staffing has been reported to cause frequent medication errors and surgical site infections as well as accidents such as bedsores and falls [33]. The International Council of Nurses stressed that providing quality nursing service by securing an appropriate nurse staffing ensures patient safety and health [34]. The number of active nurses per capita is 4.6 per 1,000 population, which substantially falls short of the OECD average of 9.3 per 1,000 population [35]. In addition to expanding nursing staffing, policy support is also needed, such as by reflecting responsibility through salary.

This study has great significance in that there are little studies examining the perception of open disclosure of PSIs among nurses and factors that affect it, and that it administered a survey on nurses in a country that currently lacks an apology law. This study has a few limitations. First, our study population consisted of nurses working in large hospitals such as general hospitals and tertiary hospitals, so our findings cannot explain PSI-related aspects among nurses in small and medium-sized hospitals and thus cannot be generalized. Second, this study is a cross-sectional survey, which limits our ability to clearly describe the causal relationships among the perception of open disclosure of PSIs, ethical awareness, and perception of patient safety culture. Thus, additional qualitative and quantitative studies are needed to substantiate the casual relationships among these factors. Third, Jang’s tool measured ethical awareness, including the contents of nurses' daily duties, responsibilities, and conflict situations. In Milliken's research, ethical awareness is a key element of ethical sensitivity and is described as something that should be done. We agree with this point and think it would have been better if we used Milliken's Ethical Awareness Scale, which has established reliability and validity.

Lastly, the true response rate might not be determined because we only included participants with passionate feelings on the topic, thus, leading to selection bias.

## Conclusions

This study found that ethical awareness and perception of patient safety culture are important in the perception of open disclosure of PSIs among nurses working in general or tertiary hospitals. Based on these results, a culture that educates, perceives, and practices the fundamental ethical values needs to be fostered with joint effort by the government, people, healthcare institutions, and healthcare professionals in order to boost nurses’ ethical awareness and perception of patient safety culture. Moreover, both hospital organizations and nurses should endeavor to implement systematic and continuous quality improvement programs, administer organization-wide education, foster a cooperative organizational culture such as teamwork, and improve staffing. Finally, an apology law should be enacted promptly such that nurses could be legally protected when who they openly disclose of a PSI based on their ethical awareness.

## Data Availability

The datasets used during the current study are available from the corresponding author on reasonable request.

## Abbreviations

PSIs: Patient safety incidents
CVI: Content validity index
AHRQ: Agency for Healthcare Research and Quality
